# Contact, agency, and haptic experience in sport: a conceptual analysis of embodied skill, interpersonal touch, and digital mediation

**DOI:** 10.3389/fpsyg.2026.1914946

**Published:** 2026-08-27

**Authors:** Yanli Tan, Xueqing Mou, Shiqi Liu, Liuhong Zang, Haiwen Cui

**Affiliations:** School of Physical Education, Xinjiang Normal University, Urumqi, China

**Keywords:** digital mediation, disability sport, embodied skill, haptic experience, pedagogical touch, sense of agency, sport, vibrotactile feedback

## Abstract

Sport is often analyzed through visual, physiological, or cognitive models, yet athletes also act through pressure, flow, friction, rebound, thermal change, bodily guidance, proximity, and vibration. These phenomena are usually examined separately in research on perceptual skill, coaching and safeguarding, disability access, and haptic technology. This conceptual analysis develops the Contact–Agency Framework to compare how body–environment, body–other, and body–technology contact reorganize opportunities to perceive, interpret, calibrate, regulate, contest, and delegate action-relevant judgment. A structured, purposive synthesis distinguishes tactile sensation, tactile perception, haptic perception, and haptic experience; treats proprioception, kinesthesia, and task-relevant thermoreception as analytically distinct but functionally coupled; and positions interoception, pain, fatigue, and affective touch as adjacent modulators. Environmental haptic information may support skilled calibration when it is detectable, discriminable, and task relevant. Interpersonal touch may guide or constrain depending on relational authorization, power, consent, and contestability. Digital mediation may expand access or displace judgment depending on mediation function, feedback schedule, interface transparency, and user control. Disability sport and Taijiquan push-hands function as analytically demanding boundary cases that expose limits of visually dominant and one-directional accounts. Five propositions specify candidate indicators, measurement designs, expected patterns, boundary conditions, and limitations. The framework is offered as a mid-level analytical heuristic, not a general theory of touch.

## 1 Introduction: why contact requires an agency framework

Sporting action is never only seen or measured. A swimmer detects pressure, flow, and temperature across the hand and forearm; a runner differentiates stable from unstable or thermally altered ground through the feet; a judoka receives information through grip, load, and resistance; a coach may guide alignment through bodily contact; and a wearable device can punctuate movement with vibration. These encounters contribute to the intelligibility of action while it is being performed.

The difficulty is conceptual rather than simply empirical. Environmental haptics is commonly framed through perception–action calibration and embodied skill (Allen-Collinson and Hockey, 2011; Heath and Carter, 2024), coach–athlete touch through pedagogy, care, safeguarding, and power (Gleaves and Lang, 2017; Ryou et al., 2025), and digital haptics through motor learning and human–computer mediation (van Breda et al., 2017; Cornelio et al., 2022). These literatures provide valuable but differently organized accounts of contact. The resulting fragmentation has a practical cost: it becomes difficult to compare who provides action-relevant information, who is authorized to interpret it, who can refuse or modify it, and where judgment remains when a contact relation is removed.

Consider a gait-correction episode. Ground reaction and shoe–surface friction provide environmental information; a coach’s hand may redirect the athlete’s posture; and a vibrating wearable may signal that a threshold has been exceeded. Ecological dynamics can explain performer–environment calibration, enactivism can explain embodied sense-making, and postphenomenology can explain technological mediation. Yet none of these perspectives, used alone, provides a common vocabulary for comparing the athlete’s interpretive authority, contestability, and allocation of judgment across all three relations. The Contact–Agency Framework addresses this specific comparative problem.

The starting point is the active-touch tradition. Gibson (1962) characterized active touch as exploration of the nearby environment through movement, in contrast to passive skin stimulation. Later work described haptic perception as an integrated system involving cutaneous, muscular, tendinous, and articular information acquired through exploratory procedures (Lederman and Klatzky, 2009). Recent scholarship similarly portrays haptic perception and action as mutually organized, with task-relevant information in water, ground, equipment, or an opponent’s body emerging through movement itself (Klatzky, 2025).

This article therefore asks: under what conditions does materially, interpersonally, or technologically mediated contact support, constrain, or redistribute a participant’s practical agency? Agency is not reduced to unrestricted choice. It concerns situated opportunities to perceive, interpret, calibrate, regulate, contest, and, where appropriate, delegate action-relevant judgment. The proposed framework is intentionally integrative but not totalizing: it preserves conceptual differences, identifies mechanisms and boundary conditions, and generates propositions that can be challenged empirically.

## 2 Conceptual scope and analytical procedure

This article uses structured conceptual analysis rather than a systematic-review design. The source corpus is purposive and problem driven: it brings together foundational and recent works that (a) define touch-related or agency-related constructs, (b) specify a relevant perceptual, relational, or technological mechanism, (c) provide a sport-specific case, or (d) reveal a boundary condition for the proposed framework. The analysis does not claim exhaustive coverage of every discipline in which touch or agency is studied.

To make the source-selection process transparent without retrospectively presenting it as a systematic review, the analysis used conceptual sampling. Candidate publications were retained only when they could perform at least one of the four analytic roles specified above: defining a construct, identifying a mechanism, supplying a sport-specific case, or exposing a boundary condition. The working corpus was expanded iteratively through targeted searches around the focal constructs and citation tracing from foundational and sport-specific sources. Sampling ceased when additional publications repeated established definitions or examples without changing the framework’s distinctions, mechanisms, propositions, or boundary conditions. The corpus included conceptual, philosophical, experimental, review, and qualitative scholarship because these designs contribute different forms of evidence to a conceptual analysis. No frequency counts, coverage estimates, or claims of representativeness are made, and the possibility of language and disciplinary bias is addressed explicitly in section “10 Limitations.”

The analysis proceeded in six linked steps. First, the phenomenon domain was delimited to bodily contact that becomes usable, interpretable, or consequential for action. Second, adjacent constructs were compared by sensory source, object of perception, role of movement, experiential level, and relation to action. Third, the literature was clustered into body–environment, body–other, and body–technology relations. Fourth, the mechanism linking each relation to agency was specified. Fifth, disability sport and sustained reciprocal contact were used as pressure tests because they expose assumptions of visual primacy and one-directional feedback. Sixth, propositions were derived only where a mechanism, boundary condition, and potential empirical indicator could be stated. This procedure follows theory-construction approaches that require an account of what is explained, by which mechanisms, and under what conditions (Borsboom et al., 2021), as well as guidance that conceptual contributions should integrate, problematize, or revise existing concepts rather than merely rename them (Jaakkola, 2020; Jabareen, 2009).

### 2.1 What counts as haptic experience?

Haptic experience is defined here as the first-person, action-relevant organization of information generated through active or receptive bodily contact with objects, environments, other persons, or technologies. It may include pressure, friction, resistance, rebound, vibration, deformation, flow, and thermal change when these relations become usable for orientation, interpretation, calibration, or regulation. For example, a swimmer may use changing hand pressure and flow to adjust propulsion, a runner may use foot–ground friction to regulate step placement, a racket-sport athlete may use rebound to time the next action, and a wearable vibration becomes action relevant only when it is interpreted as a cue. The definition accommodates involuntary, unpleasant, and harmful contact as well as voluntary and beneficial contact.

The term is intentionally broader than tactile sensation but narrower than general bodily experience. Tactile sensation refers to the registration of cutaneous and thermal stimulation at or near the body surface. Tactile perception refers to the organization and discrimination of those skin-based signals. Haptic perception refers to object-, environment-, person-, or device-relevant perception through contact, commonly integrating cutaneous, thermal, proprioceptive, and motor information. Haptic experience denotes how such contact is lived and used in an action context. These levels overlap in practice, but they should not be used as synonyms.

Thermal information is included because temperature and heat transfer can alter both what is detected and how contact is interpreted. Non-noxious warming can change vibrotactile detection and discrimination, demonstrating interaction rather than independence between thermal and tactile processing (Zhang et al., 2009). In sport, water temperature, surface temperature, equipment heat, and cold-induced changes in sensitivity may therefore become task relevant without making all thermoreception haptic by definition.

Proprioception remains analytically distinct but functionally coupled. It concerns body position, movement, and force, whereas haptic perception concerns relations between an acting body and contacted objects, surfaces, persons, or devices (Proske and Gandevia, 2012). Kinesthesia is used here for the movement-related component of proprioception rather than as a separate sensory modality; no theoretical claim in this article depends on treating the two terms as independent systems. A tennis player may sense elbow angle proprioceptively while reading racket resistance and rebound haptically. In active touch, these systems cooperate; conceptual distinction does not imply physiological or practical separation.

Affective touch is also adjacent but not identical. Research distinguishes discriminative touch, which supports localization and object-related information, from touch that carries social-affective meaning (McGlone et al., 2014). The same coach–athlete contact may therefore be technically informative, emotionally reassuring, socially ambiguous, or ethically inappropriate. Interoception, pain, fatigue, and emotion are treated as neighboring systems that modulate contact interpretation, willingness to explore, and capacity to regulate action rather than as constituents of haptic experience in every case.

Dynamic touch provides an important bridge. It describes how people acquire information about objects through wielding, holding, or moving them so that inertia, length, or resistance becomes perceptually available in action (Travieso et al., 2020). For athletes, a bat, paddle, shoe, rope, bicycle, or wheelchair can participate in a skilled action system without becoming a literal body part.

### 2.2 Agency, autonomy, control, and interpretive authority

Agency refers here to situated opportunities and capacities to initiate, monitor, interpret, calibrate, regulate, contest, or delegate action-relevant judgment. Sense of agency is the experience of authorship or control over an action and its effects (Haggard, 2017). Control refers more narrowly to the actual or perceived capacity to alter an action process or outcome. Autonomy is a normative concept concerning whether action is self-endorsed and responsive to the participant’s values and reasons; in exercise contexts, autonomy-supportive conditions are associated with sustained, self-regulated participation (Teixeira et al., 2012). Interpretive authority concerns whose account of bodily signals, contact meaning, or required correction is treated as decisive. Embodied agency denotes agency realized through bodily skills, sensory access, and perception–action relations rather than through disembodied choice alone.

These distinctions matter because a contact relation may improve immediate performance while reducing autonomy, may preserve autonomy while offering little effective control, or may distribute control across athlete and device without eliminating agency. The framework therefore does not classify contact as beneficial or harmful solely from movement accuracy. It asks how information, decision rights, contestability, and dependence are organized. The conceptual boundaries and working definitions used in the framework are summarized in Table 1.

**TABLE 1:** Conceptual boundaries used in the Contact–Agency Framework.

Construct	Working definition	Relationship to the framework	Boundary rule	Sport-specific example and agency implication
Tactile sensation	Registration of cutaneous and thermal stimulation at or near the body surface.	Potential sensory input to haptic experience.	Does not by itself imply object/environment interpretation or action use.	A runner feels a shoe vibration. It affects agency only if the signal can be distinguished and related to a task.
Tactile perception	Organization and discrimination of skin-based signals.	Supports localization, intensity, texture, and temperature judgments.	May occur without active exploration or broader action meaning.	An athlete identifies the location or intensity of a vibration, supporting cue localization but not yet task interpretation.
Haptic perception	Perception of objects, environments, persons, or devices through contact, often integrating cutaneous, thermal, proprioceptive, and motor information.	Perceptual process central to body–environment, body–other, and body–technology relations.	Defined by contact-relevant object or relational information, not general bodily feeling.	A swimmer reads hand pressure and flow to alter stroke orientation, supporting skilled calibration.
Haptic experience	First-person, action-relevant organization of contact information and meaning.	Primary experiential phenomenon analyzed.	Includes harmful and involuntary contact; excludes bodily states unrelated to contact.	A judoka experiences grip and load as an opening, threat, or constraint, shaping action and judgment.
Proprioception / kinesthesia	Sensing body position, movement, and force.	Analytically distinct but commonly coupled with haptic perception.	Primarily tracks the body’s mechanical state rather than the contacted relation.	A tennis player senses elbow angle while haptically reading racket rebound; both contribute to self-calibration.
Thermoreception	Sensing temperature and thermal change.	Can contribute to haptic experience when contact temperature is task relevant.	Not every thermal experience is haptic; noxious heat may additionally involve pain.	Water or surface temperature may alter exploration, grip, pacing, and safety judgments.
Affective touch	Social-emotional and regulatory meaning of interpersonal contact.	Overlaps with pedagogical touch and relational authorization.	Affective meaning does not establish technical usefulness or ethical authorization.	A coach’s hand may reassure or intrude; agency depends on consent, power, and contestability.
Interoception / pain / fatigue	Internal physiological, nociceptive, or effort-related information.	Modulates contact interpretation and action capacity.	Does not independently establish contact-based perception.	Fatigue may raise discrimination thresholds, making previously useful contact feel ambiguous or overwhelming.
Sense of agency	Experience of authorship or control over action and effects.	One possible agency outcome.	Not equivalent to autonomy, objective control, or performance accuracy.	An athlete may improve after a prompt yet attribute correction to the device rather than to their own action.
Autonomy / control / interpretive authority	Self-endorsed action; capacity to alter action; standing to define bodily meaning.	Distinct dimensions used to analyze contact consequences.	May diverge within the same contact event.	An athlete may refuse coach touch or override a wearable even when performance gains are possible.

### 2.3 Theoretical position and added value

The Contact–Agency Framework does not replace ecological dynamics, enactivism, phenomenology, sense-of-agency research, or postphenomenology. It uses them as complementary resources while addressing a narrower comparative problem. Ecological dynamics clarifies performer–environment calibration; enactivism emphasizes embodied sense-making and relational agency arising through organism–environment engagement (Popova and Ra̧czaszek-Leonardi, 2020; Segundo-Ortin, 2020); phenomenology describes lived bodily experience; sense-of-agency research clarifies action authorship and control; and postphenomenology analyzes how technologies mediate perception and action by amplifying some relations while reducing or transforming others (Ihde, 1990; Verbeek, 2005). The added value is a common set of questions that can be applied across environmental, interpersonal, and technological contact without collapsing their differences: What information is available? Who may interpret it? Who sets the correction threshold? Can the participant contest or modify the relation? What remains possible when contact is removed?

Importantly, the framework does not fuse these traditions into a single theory. It treats them as addressing different explanatory levels and places their insights in a comparative matrix. A single sport episode may involve ecological calibration to a surface, an interpersonal negotiation of permission and authority, and a technologically mediated threshold that redirects attention. Existing perspectives can illuminate each layer, but their units of analysis and normative commitments differ. The framework’s contribution is therefore relational and mid-level: it makes the allocation of information, interpretation, contestability, and decision rights comparable across contact relations while retaining the distinct mechanisms appropriate to each domain. The relationship between the Contact–Agency Framework and these adjacent theoretical perspectives is summarized in Table 2. This is what permits the gait-correction example to be analyzed as one episode without implying that ground friction, a coach’s hand, and an algorithmic vibration are phenomenologically, functionally, or ethically equivalent.

**TABLE 2:** Relationship of the Contact–Agency Framework to adjacent theoretical perspectives.

Perspective	Central contribution	What remains unresolved for this article’s problem	Contact–Agency contribution
Ecological dynamics	Performer–environment constraints, affordances, perception–action calibration.	Limited common vocabulary for consent, interpersonal authority, and algorithmic allocation of judgment.	Extends relational analysis across environmental, interpersonal, and technological contact.
Enactivism / participatory sense-making	Embodied sense-making, autonomy, and interactional coordination.	Does not by itself specify measurable contact categories, feedback schedules, or interface control.	Links sense-making to contact-specific mechanisms, indicators, and boundary conditions.
Phenomenology / sense-of-agency research	Lived bodily experience, action authorship, prediction, and control.	First-person experience may remain disconnected from institutional power, design, and observable coordination.	Connects reported experience with relational conditions and empirical measures.
Postphenomenology / technology mediation	Technology shapes perception, action, and relations to the world.	Often treats technology separately from sport-specific environmental skill and pedagogical touch.	Compares body–technology mediation with body–environment and body–other relations using shared agency questions.

The framework also limits its own scope. It applies when bodily contact is action relevant and when the distribution of judgment is analytically consequential; it is not intended to subsume all multisensory experience, all social interaction, or all technology use. Nor does it assume that agency always increases with choice or decreases with delegation. In some disability-sport settings, stable delegation to a guide or device may enlarge practical agency; in high-risk coaching, temporary restriction may be justified while still requiring intelligibility, proportionality, and contestability. These counterexamples prevent agency from becoming a universal positive label and make the framework’s propositions open to empirical challenge.

## 3 The Contact–Agency Framework

**FIGURE 1:**
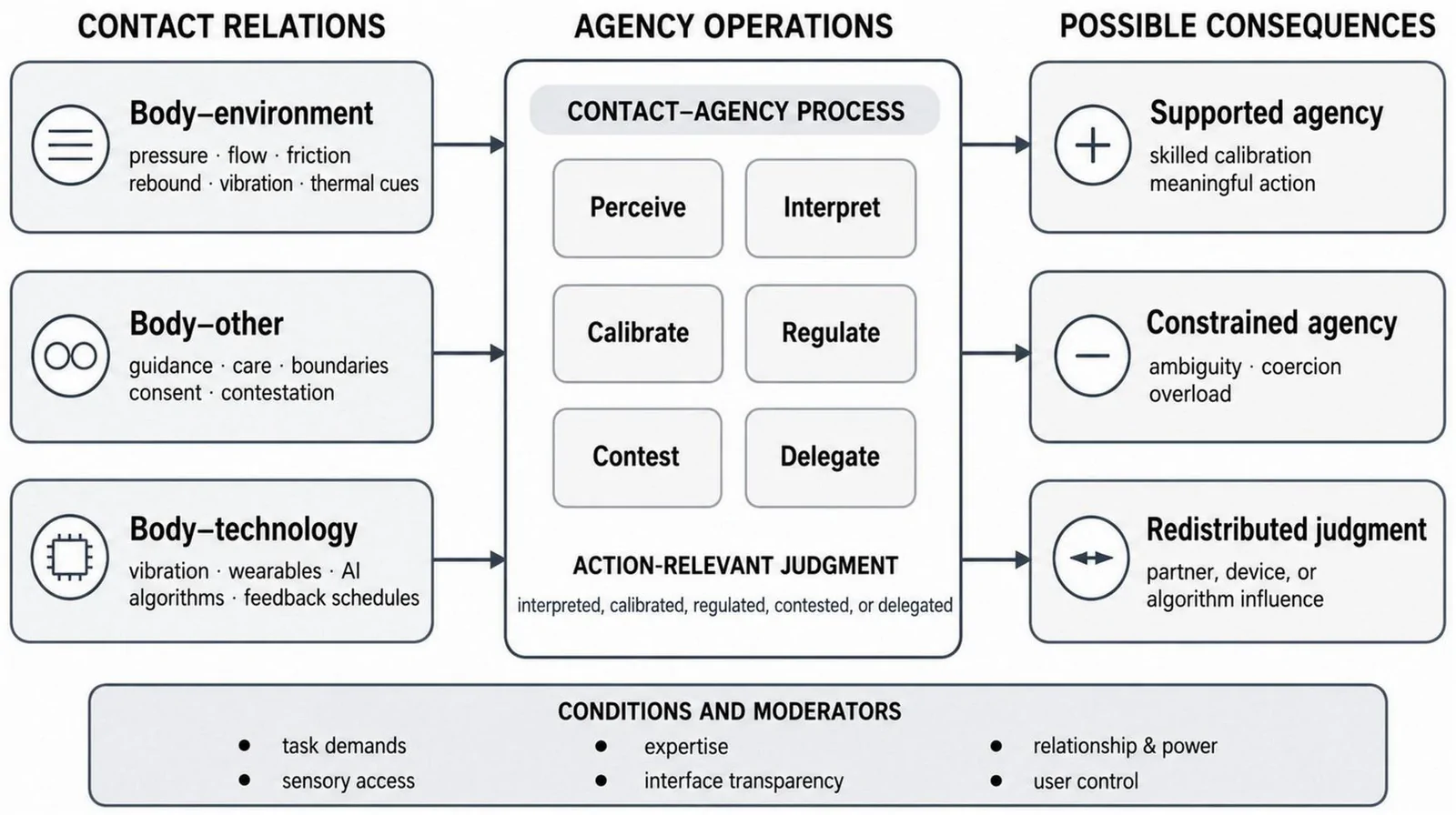
The Contact–Agency Framework. The model is analytical rather than deterministic: the same contact can support, constrain, or redistribute agency depending on task, expertise, relationship and power, sensory access, interface transparency, and user control.

The framework separates contact relations, agency operations, and possible consequences. Contact relations identify where and through whom or what contact occurs: body–environment, body–other, or body–technology. Agency operations identify what participants can do with contact-relevant information: perceive, interpret, calibrate, regulate, contest, or delegate judgment. Possible consequences describe contingent changes in practical agency: support, constraint, or redistribution. These levels are not interchangeable. The three contact relations, their primary mechanisms, and their associated agency questions are summarized in Table 3. A body–technology relation, for example, may support perception while constraining interpretation if an opaque algorithm fixes the meaning of the cue. The arrows in Figure 1 therefore indicate analytic pathways, not deterministic or one-to-one causal effects.

**TABLE 3:** Contact relations, mechanisms, and agency questions.

Contact relation	Contact information or event	Primary mechanism	Agency question
Body–environment	Pressure, flow, friction, rebound, vibration, deformation, surface, temperature.	Detectability, discrimination, exploration, and skilled calibration.	Does contact enable task-relevant interpretation, or do masking, overload, and instability obscure it?
Body–other	Guidance, protection, correction, reassurance, restraint, proximity.	Relational authorization, consent, trust, contestability, boundary recognition.	Can the athlete understand, refuse, modify, and participate in the contact relation?
Body–technology	Vibration prompts, smart garments, wearables, VR/AR interfaces, sensor and AI alerts.	Mediation function, feedback schedule, transparency, and allocation of control.	Does the system expand sensory access and interpretation, or retain judgment in opaque thresholds and prompts?

The framework is analytical rather than deterministic. The same contact may support agency by enabling skilled calibration, constrain agency through ambiguity, coercion, or overload, or redistribute judgment across partners and technologies. Outcomes depend on task demands, expertise, sensory access, relationship and power, cultural context, interface design, transparency, and user control.

## 4 Body–environment contact: environmental haptic information and skilled calibration

A body–environment account treats skilled movement as an adaptive organization of performer, task, and environmental constraints. Ecological dynamics describes expert performance in relational terms (Seifert et al., 2013), and environmental design has been shown to shape perceptual–motor exploration, learning, and transfer in contact-intensive tasks such as climbing (Seifert et al., 2015). Enactive accounts likewise emphasize that athletes learn workable relations within a changing field of possibilities rather than merely reproducing form (Avilés et al., 2020).

Haptic experience becomes especially visible when an environment resists, yields, flows, vibrates, rebounds, changes temperature, or changes direction. Distance runners and scuba divers have described how ground texture, pressure, temperature, air, and water become meaningful through ongoing movement rather than as detached sensory variables (Allen-Collinson and Hockey, 2011). Swimming is especially instructive because pressure, flow, buoyancy, drag, and thermal conditions continuously change around the athlete. Ethnographic work describes “feel for the water” as cultivated through repeated engagement and as learned, transmitted, embodied knowledge (Heath, 2022; Heath and Carter, 2024).

The relevant category is therefore environmental haptic information, not material information alone. Material properties such as texture, friction, stiffness, compliance, and heat transfer are important in running or climbing, but swimming additionally depends on fluid pressure, flow, drag, and turbulence. The broader term preserves the task-relevant relation while avoiding a surface-only account.

Contact information also operates at collective scales. In rugby union scrums, pack force depends not only on individual strength but on body position, force transmission, and coordination as a unit (Quarrie and Wilson, 2000). In coxless-pair rowing, athletes coordinate oar angles and forces and may experience their partner through changes in boat behavior; variability can be functional or perturbing depending on boat velocity and whether the crew shares the same interpretation of the adjustment (Seifert et al., 2017). These cases extend the argument beyond individual sports: the relevant unit of haptic calibration may be a dyad, pack, or crew whose members jointly produce and interpret contact information.

The distinction between information and noise must also be operational rather than metaphorical. Contact becomes usable information when stimulus differences are detectable and discriminable relative to background stimulation and task demands. Candidate psychophysical parameters include absolute detection thresholds, difference thresholds or just-noticeable differences, temporal and spatial resolution, signal-to-noise ratio, and masking effects. Experimental work shows that an additional mechanical vibration can elevate detection thresholds for another tactile signal, particularly when frequencies are similar or masking intensity increases (Ryu et al., 2018). Thus, “noise” is not an intrinsic property of a stimulus: it is a relation among signal characteristics, competing stimulation, sensory channel, expertise, fatigue, and task goal.

P1. Environmental haptic information is more likely to support skilled calibration when task-relevant variations in pressure, flow, friction, rebound, vibration, deformation, or thermal change are detectable, discriminable, and interpretable within a sport-specific perception–action system. The same stimulation is more likely to constrain performance when masking, overload, instability, fatigue, or insufficient skill prevents reliable discrimination.

P1 can be examined through expert–novice comparisons; manipulated surfaces, water flow, equipment, resistance, temperature, or vibration; psychophysical threshold procedures; and combined movement and elicitation data. Candidate indicators include detection threshold, just-noticeable difference, discrimination accuracy, adaptation rate, error correction latency, performance under masking, and retention or transfer after altered contact conditions.

## 5 Body–other contact: pedagogical touch, consent, and relational authorization

Interpersonal touch creates a different theoretical problem. A coach may place a hand on an athlete’s shoulder, pelvis, arm, or back to guide alignment, prevent injury, calm distress, or facilitate correction. Physical features alone do not settle the meaning of contact. Relationship, role, purpose, timing, bodily location, explanation, cultural convention, and the participant’s ability to accept, question, or refuse all shape interpretation.

Direct sport evidence indicates that pedagogical touch can be part of coach–athlete sensory interaction. A sensory ethnography of high-performance coaching showed how touch can communicate body form, explore movement qualities, and coordinate instruction across sport settings (Ryou et al., 2025). Broader interpersonal-touch research identifies care, communication, affiliation, regulation, hierarchy, and power as possible functions (Gallace and Spence, 2010; Saluja et al., 2024). Sport scholarship simultaneously warns that blanket no-touch discourses may narrow legitimate coaching care and safety practices, while visual and sociological analyses situate touch within governance and power (Gleaves and Lang, 2017; Jones et al., 2013).

Safeguarding is therefore not an external add-on. Consent in coach–athlete relations is complicated by authority, dependence, age, selection pressure, performance expectations, and organizational culture (Johansson, 2013). Power imbalance, boundary ambiguity, and institutional conditions are documented risk factors in research on sexual violence in coach–athlete relationships, although those findings should not be generalized to every nonsexual contact event (Gaedicke et al., 2021).

These concerns intensify for minors and for athletes whose participation depends on coaches, guides, assistants, or personal-care support. Experimental evidence indicates that coercive instruction can reduce the subjective sense of agency (Caspar et al., 2016), so apparent compliance should not be equated with authorization. For child and adolescent athletes, developmental capacity, parental delegation, selection pressure, and long-term dependence on coaches raise the required standard for explanation, proportionate purpose, non-contact alternatives, documented safeguards, and independent reporting routes (Gleaves and Lang, 2017). Comparable safeguards are necessary in disability sport where routine assistance may normalize contact that still requires continuing negotiation.

Relational authorization refers to the situated process through which contact becomes intelligible and contestable as guidance, care, correction, protection, control, or intrusion. It extends beyond one-time verbal permission. Relevant conditions include a clear and proportionate purpose, communicative preparation, sensitivity to body location and vulnerability, available non-contact alternatives, the possibility of refusal without penalty, ongoing checking, relational history, and institutional safeguards. Interpretive authority should not default to the coach merely because the contact appears technically effective.

P2. Coach–athlete contact is more likely to support pedagogical and embodied agency when its purpose, location, duration, alternatives, and limits are intelligible; when refusal or modification is practically possible; and when power asymmetries are actively managed. The same physical contact is more likely to constrain agency when it is ambiguous, coercive, normalized without review, or insulated from contestation.

P2 can be studied through video-stimulated recall, dyadic interviews, event-level observational coding, and divergence between coach and athlete accounts. Candidate indicators include perceived appropriateness, trust, perceived autonomy, body-related safety, refusal efficacy, explanation quality, agreement about purpose, and whether performance benefits persist when physical guidance is withdrawn.

## 6 Body–technology contact: mediation function, feedback schedule, and user control

Digital systems make contact both deliberate and potentially opaque. Wearables, smart garments, vibrating watches, instrumented footwear, virtual-reality devices, and haptic interfaces translate movement, posture, pace, impact, or physiological thresholds into tactile prompts. These cues may reduce visual load and provide timely information, but they can also insert device classifications and algorithmic thresholds between athletes and their own bodily judgment.

Motor-learning research shows that augmented visual, auditory, haptic, and multimodal feedback can support learning under particular task, timing, complexity, and learner conditions (Sigrist et al., 2013). Reviews of vibrotactile feedback in motor learning and sport similarly report promising but heterogeneous effects that depend on task type, cue placement, feedback schedule, and training duration (van Breda et al., 2017). Sport-specific haptic feedback can alter gait-related outcomes, yet immediate improvement does not establish stronger agency or independent interpretation after the device is removed (Sheerin et al., 2020). Self-controlled haptic assistance research also suggests that user choice, feedback frequency, and how assistance is understood can influence retention and dependence (Williams et al., 2017).

Three dimensions must be distinguished. Mediation function concerns whether feedback supports perception of information the athlete can increasingly use independently or substitutes for information that remains unavailable without the device. Feedback schedule concerns whether cues are concurrent, fixed, reduced, faded, intermittent, or adaptive. Interface control concerns whether thresholds and cue parameters are system set, shared, or user adjustable. A faded schedule is not equivalent to user control, and substitutive feedback is not inherently agency reducing: in disability sport, stable substitution may be an enabling condition rather than a temporary scaffold.

P3. Digital haptic mediation is more likely to support embodied agency when its function and schedule preserve exploration, develop interpretable links to bodily or environmental information, and permit retention or transfer where independent performance is an appropriate goal. It is more likely to create dependency when adjustment remains tied to opaque, externally prescribed cues without opportunities to understand or operate beyond them. Stable substitutive feedback may nevertheless support agency when it provides otherwise inaccessible task information and remains aligned with the participant’s goals.

P4. Greater interface transparency and practical ability to modify, pause, reject, or reinterpret haptic prompts are expected to support perceived and practical agency, provided that safety-critical constraints and the consequences of override are explicit. User control should therefore be analyzed separately from feedback fading and from the supportive or substitutive function of the device.

AI-driven feedback intensifies these issues. Algorithms may personalize thresholds and timing, but personalization does not by itself guarantee transparency or user autonomy. Athletes should be able to understand the basis and limits of recommendations at an appropriate level, know what data are collected and retained, identify whether coaches or organizations can access them, and challenge or override outputs when feasible. Relevant risks include privacy loss, biased calibration, false precision, automated escalation of performance norms, and overreliance that weakens attention to intrinsic cues. Biometric and physiological data require explicit governance because they may be identifying and may be repurposed beyond immediate feedback. Sensor drift, calibration loss, latency, malfunction, or device failure can generate erroneous prompts; meaningful human oversight should therefore allow athletes and qualified staff to review, contest, suspend, or override system outputs. These issues are consistent with evidence that interface design can preserve or erode the sense of agency in human–computer integration (Cornelio et al., 2022), as well as critiques of imposed monitoring and dataveillance (Lupton, 2016) and autonomy concerns surrounding health-promoting wearables (Owens and Cribb, 2019).

## 7 Pressure tests

### 7.1 Disability sport and sensory access

Disability sport is a central pressure test because it reveals how sport theory may assume visual observation as the default route to competence. For athletes with visual impairments, participation is shaped by instruction, environmental legibility, adapted materials, knowledgeable partners, and meaningful sensory cues (Haegele and Porretta, 2015). Research on judo indicates that vision remains relevant even in a sport beginning with continuous grip contact, underscoring that competence is multisensory and task specific (Krabben et al., 2018). Tactile and auditory adaptations can improve immediate performance under particular test conditions, but such findings do not establish complete sensory compensation or long-term agency (Kons et al., 2023).

The framework therefore treats sensory access as constitutive of competence. An environment is not accessible merely because entry is permitted; task-relevant information must be detectable, interpretable, actionable, and, where appropriate, adjustable for participants with different sensory orientations. Substitutive technologies or partner contact may enhance agency when they expand access and choice rather than enforcing a single normative sensory pathway.

### 7.2 Taijiquan push-hands and reciprocal haptic attunement

Taijiquan push-hands provides a second analytically demanding boundary case because contact is sustained, mutual, and dynamically consequential. Movement-studies scholarship on Aikido, Taijiquan-tuishou, and Contact Improvisation shows how sustained touch can coordinate balance, weight transfer, direction, and movement between bodies (Gómez-Lozano et al., 2022). The traditional term ting jin is not treated as an internationally settled psychological construct. Instead, reciprocal haptic attunement is used provisionally for ongoing mutual adjustment of force, timing, posture, pressure, and balance through sustained contact.

Direct biomechanical studies provide a closer sport-specific basis. In a case study, an experienced practitioner redirected incoming force through subtle postural adjustment rather than rigid resistance, as shown by motion analysis, electromyography, and force-plate data (Chen et al., 2010). A study of 10 practitioners found that resultant ground-reaction force remained close to body weight across attack and defense phases, consistent with continuous force redistribution rather than simple maximal pushing (Chang et al., 2014). These studies do not validate ting jin as a unitary psychological construct, but they demonstrate that Push Hands involves measurable regulation of force direction, posture, balance, and partner-induced perturbation.

The concept is informed by participatory sense-making, in which social understanding emerges through coordinated interaction rather than within isolated individuals (Fuchs and De Jaegher, 2009). It prevents one-directional feedback from becoming the default model of sport haptics: each movement perturbs and reorganizes the field of possible movement for the other. Ethnographic work on boxing similarly emphasizes bodily apprenticeship, dependence, and practical adjustment rather than disembodied rule learning (Wacquant, 2004).

P5. In sustained-contact practices, reciprocal haptic attunement is more likely to support coordinated agency when partners continuously and responsively adjust force, timing, posture, and balance; it is more likely to weaken when contact becomes unilateral imposition, rigid resistance, or unresponsive compliance.

P5 should be tested with indicators rather than tools alone. Candidate indicators include mean and peak interaction force, force variability, time-lagged cross-correlation between partner kinematics, lag symmetry, relative-phase stability, center-of-pressure coupling, recovery latency after perturbation, adaptation across trials, and perceived co-control. Dual motion capture, force sensing, synchronized electromyography, and post-task elicitation interviews are methods for obtaining these indicators, not indicators themselves.

## 8 Operationalization and research agenda

The propositions are not universal laws. Each links a contact relation to a mechanism, candidate indicators, and boundary conditions. Multi-method designs are especially important because movement improvement, subjective authorship, autonomy, and interpretive authority may diverge. The five propositions, candidate indicators, measurement approaches, and principal boundary conditions are summarized in Table 4. Movement capture and force data can identify coordination changes; psychophysical procedures can establish detectability and discrimination; stimulated recall can examine how a cue or touch was interpreted; interface logs can reveal modification, rejection, or reliance; and retention or transfer tests can assess what remains when assistance is removed.

**TABLE 4:** Propositions, indicators, methods, and boundary conditions for testing the Contact–Agency Framework.

Proposition	Mechanism and expected pattern	Candidate variables / indicators	Measurement tools / design	Boundary conditions and principal limitations
P1 Environmental haptic information	Detectable and discriminable task-relevant contact should predict lower calibration error, faster adaptation, and better retention; masking or overload should reverse this pattern.	Detection/JND; discrimination accuracy; masking cost; error-correction latency; adaptation; retention/transfer.	Psychophysics; expert–novice comparisons; manipulated surface/flow/temperature/ vibration; movement data; elicitation.	Expertise; fatigue; task stability; competing stimulation; sensory access. Threshold tasks may not generalize directly to competition.
P2 Relational authorization	Authorized and contestable touch should predict greater safety, autonomy, and coach–athlete agreement; ambiguous or coercive touch should not.	Appropriateness; trust; autonomy; safety; refusal efficacy; coach–athlete agreement; persistence after withdrawal.	Video-stimulated recall; dyadic interviews; event coding; questionnaires.	Minor status; dependency; power; culture; body location; stakes; alternatives. Self-report is vulnerable to social desirability and role pressure.
P3 Mediation function and schedule	Supportive or necessary substitutive cues should improve access; faded support should improve transfer where independence is appropriate, whereas opaque fixed dependence should reduce it.	Retention/transfer without device; reliance; cue-response accuracy; intrinsic-cue detection; performance after cue removal.	Concurrent/faded/adaptive comparisons; accessibility studies; device-removal tests; qualitative accounts.	Disability/access need; task risk; skill; frequency; reliability. Performance gain alone does not establish agency, and cue removal may be inappropriate.
P4 Transparency and user control	Transparency and practical control should predict calibrated trust, appropriate modification, and justified override rather than indiscriminate compliance or rejection.	Override rate; parameter changes; comprehension; perceived control; trust calibration; disagreement with system.	User-control experiments; interface logs; interviews; scenario and failure-mode testing.	Safety constraints; authority; stakes; opacity; biometric governance; oversight. Logs cannot fully explain why users complied or resisted.
P5 Reciprocal haptic attunement	Responsive mutual adjustment should predict stable relative phase, lower recovery latency, and greater co-control; unilateral imposition should weaken coupling.	Mean/peak force; force variability; cross-lag; lag symmetry; relative phase; COP coupling; recovery latency; co-control.	Dual motion capture; force sensing; synchronized EMG; paired interviews.	Cooperative/competitive framing; expertise; duration; asymmetry; rules. Small dyadic studies may limit generalizability.

A strong empirical program should predefine which dimension of agency is being tested. Sense of agency may be measured through authorship or control judgments; practical control through successful modification or override; autonomy through self-endorsement and absence of coercion; interpretive authority through whose account guides decisions; and embodied agency through the capacity to use sensory information adaptively. Treating these outcomes as interchangeable would recreate the conceptual ambiguity the framework is intended to reduce.

## 9 Ethical, governance, and design implications

For coaching, the framework implies that technically effective touch is not automatically ethically authorized. Organizations should specify when physical guidance is necessary, how consent is obtained and renewed, how power asymmetries are addressed, and how athletes can refuse or report contact without jeopardizing selection or participation. Safeguarding should protect against coercion while preserving proportionate, explainable, and contestable forms of care and instruction.

For digital systems, agency-sensitive design requires more than accuracy. Interfaces should disclose cue logic at an appropriate level, provide meaningful control where possible, distinguish supportive from substitutive functions, document feedback schedules, and test retention, transfer, and dependency. Data governance should clarify collection, access, storage, secondary use, deletion, and the specific handling of biometric and physiological data. Systems should document foreseeable failure modes, support meaningful human review, and specify how athletes and coaches may disregard, suspend, or override an erroneous prompt without penalty. AI-driven systems should not present probabilistic outputs as unquestionable bodily truth.

For disability sport, design should begin from sensory access and participant goals rather than from restoration of a presumed normal modality. Stable partner or technological mediation may be agency enabling when it expands action possibilities and remains accountable to the athlete.

## 10 Limitations

Several limitations constrain the proposal. First, the purposive conceptual corpus is not exhaustive and may underrepresent non-English scholarship, traditions outside the selected disciplines, and sport practices in which contact has different cultural meanings. Second, the distinctions are analytical; tactile, proprioceptive, thermal, affective, painful, and interoceptive dimensions may be inseparable in lived experience. The focal constructs also carry different meanings across psychophysics, motor control, phenomenology, sociology, and science and technology studies; the working definitions in Table 1 are stipulative for this framework rather than field-wide settlements. Third, the framework is not a validated causal model, and the propositions require testing across sports, skill levels, ages, genders, disability types, and cultural settings.

Fourth, agency is multidimensional. Performance, perceived control, autonomy, interpretive authority, and long-term independence may not converge, and available measures may capture only part of the construct. Fifth, the two pressure tests are strategically selected rather than representative of all disability or contact sports. Sixth, digital technologies, data practices, and AI systems change rapidly, so claims about transparency, privacy, and dependence require continued empirical and normative review.

## 11 Conclusion

This conceptual analysis reframes sport haptics as a problem of contact and agency. Haptic experience is distinguished from tactile sensation and tactile perception, while proprioception and thermoreception are treated as analytically distinct but functionally coupled contributors in many contact tasks. Interoception, pain, fatigue, and affective touch remain adjacent systems that can modulate how contact is experienced and acted upon.

The Contact–Agency Framework contributes a mid-level analytical vocabulary for comparing body–environment, body–other, and body–technology relations. Its central question is not whether contact is inherently good or bad, but how a contact relation changes the participant’s opportunities to perceive, interpret, calibrate, regulate, contest, and delegate action-relevant judgment. By specifying mechanisms, indicators, boundary conditions, ethical requirements, and design implications, the framework offers a provisional basis for empirical research without claiming to unify all forms of touch or agency.
